# Treatment of the Dental Pulp

**Published:** 1879-07

**Authors:** Arthur M. Rice


AMERICAN JOURNAL

OF

DENTAL SCIENCE.

Vol. XIII. THIRD SERIES—JULY, 1879. No. 3.

### ARTICLE I.

*Treatment of the Dental Pulp.*—(Continued.)

BY ARTHUR M. RICE, D. D. S.

Some slight irritation is generally necessary to induce healthy granulation and a healing of the parts by "first intention," as it is termed, but any substance in contact with an organ so highly sensitive as the tooth-pulp, will act sufficiently to produce this effect, and any material that is an irritant in itself is likely in time to produce bad results; so we cannot use too much care in all operations of this nature.

It has now become a general practice to use some intervening substance where oxy-chloride of zinc is employed as a capping over an exposed pulp, and one of the very best materials used for this purpose is a solution of gutta-percha in chloroform. The kind used for base plates is preferred by some, owing to the color, as we can see how much is applied; other materials are also recommended, as Glycerine, Balsam of Fir, Copal Varnish in Ether, or Collodion: any of these preparations will prove serviceable and efficient as a non-irritant.

Perhaps the best method known at the present time, and one likely to be attended by the best results, consists in the use of a pad, "so to speak," composed of lacto-phosphate of lime, or oxide of zinc. Where this latter substance is used it is prepared by mixing the powder with diluted creosote or oil of cloves, using but a small quantity of either liquid. This mixture can be easily worked into a mass fitly called a pad, and is applied directly to the pulp as a covering, and then the oxy-chloride placed over it, and by this method it is claimed that the therapeutic action of the latter is not prevented but only modified by the Oxide of Zinc pad. This is evident from the fact that the powder composing the pad becomes in a short time consolidated with the Oxy-Chloride with which it is covered. In following this course but little, "if any," pain is likely to attend the operation, and it is considered one of the best and surest ways that have been tried.

The lacto-phosphate is used in the same manner, a paste is made by mixing lacto and magna enough to make a solution and then add dry precipitate to make a paste. This can be applied directly to the pulp, but it may be better to use a little of the gutta-percha and chloroform; a drop can be placed over the exposed surface with the point of an instrument, or on a piece of linen or paper cut in a circular form, and placed in such a manner that there will be but little pressure. The paste is now put in and covered with Oxy-Chloride, remembering to use some sedative like chloroform, acetate of morphia, or an emollient such as glycerine before any capping is applied, and if we are careful to observe these little points we will find our efforts crowned with success in a majority of cases.

Weston's dental caps are also recommended as effectual in capping pulps. And one great advantage possessed by them consists in the fact that, no matter what material may be used it is held in position by the cap and does not racture by the pressure used in introducing a filling. In this way Oxy-Chloride can be used in small quantities and the Chloride largely diluted, and although this preparation may not set firmly in a few minutes, it is secured in its place by the cap. These caps are made of platinum, concave, and provided with a lip on the side so that they can be conveniently handled. The concave surface is filled with the paste of whatever material is used, and pressed to its place with pliers made for the purpose. The paste has a tendency to adhere to the dentine and the cap to the paste, thus proving a protection to the pulp from mechanical pressure in the operation of filling the tooth. The principle seems a good one, and, if what is claimed is practicable they are certainly worth a trial.

When inflammation is present in the pulp a different course of treatment is adopted. Our first efforts should be directed toward establishing a healthy action, and this can only be accomplished by a careful removal of all that serves as an irritant, and by the use of such medicines as the case may require.

Acute inflammation can be distinguished from the chronic form by its symptoms, such as a gnawing pain in its first stages, which gradually increases and becomes constant, deep seated, and throbbing, and after a time almost insupportable, and is owing to the distention of the capillaries of the pulp, which crowd against the unyielding walls in which it is enclosed, and press on the nervous filaments which are everywhere distributed upon it. The course recommended where the vessels are in this congested state, is to deplete the pulp by pricking in that portion where one of the principal arteries would be most likely to be punctured, relieving the congested condition of the capillaries by the abstraction of blood, and at the same time prescribe a cathartic, or, perhaps the bromide of potassium, in doses of from 5 to 40 grains, together with a hot foot bath, and a mustard plaster at the back of the neck. Carbolic or chromic acid is then applied in small quantities and repeated often until a healthy action is set up and the parts restored to a normal condition. If we can accomplish this important result, the tooth may then be filled as in other cases of exposure.

The chronic or local form of inflammation differs from the acute, in that the pain accompanying it is periodical in its nature, occurring at irregular and uncertain intervals, and is often relieved in its early stages by local applications, but where long continued, it gives rise to ulceration of the surface of the pulp. The course of treatment here would consist in the use of Tincture of Aconite Root, Iodine, and Laudanum in equal parts, or a Nitrate of Silver solution, as our object is, to use such remedies as will bring about the formation of healthy granulations on the surface of the pulp, and a healing of the parts by first intention. Where there has been sloughing of the parts there is loss of substance and no secondary dentine will take place, although it is claimed by some that one-half of the whole substance of the pulp can be removed and the remaining part restored to health. But this is extremely doubtful. In any case of this sloughing, the proper course to pursue would be to cleanse the cavity with tepid water, then use lead water, or pure carbolic acid combined with Thymol. After the use of either of these agents, pepsin is applied. This material has a strong affinity for dead matter without any effect on the living tissue, and consequently it is considered the best medicine to be used in this condition of the pulp, and if we can restore healthy action, of course the tooth can be filled successfully. But as a general rule, our safest plan will be to devitalize and remove the pulp, and fill the chamber carefully where there is much complication in the disease.

The other diseases of the pulp, Ossification, and Fungus growth, are hardly to be considered under the head of conservative treatment, as it is necessary, "with rarely an exception," in both conditions, to destroy the pulp and fill the canal, or, in aggravated cases to extract the teeth affected with these diseases, as it would be useless to attempt any treatment with a view to preserving the vitality of the pulp.

There are certain precautions which should be observed in order to secure success in any method of capping, and even then, under some circumstances, any attempt to save a pulp after it has become exposed, is almost if not utterly useless. For instance; in the case of a person in very poor health, whose blood is thin, pale and of a serous nature, not possessing the necessary salts, &c., for forming bone; or where there is a diseased state of the gums, owing to various constitutional disorders, under such circumstances a pulp would die almost spontaneously, so it is not advisable to attempt to treat it, as we know there is scarcely any chance for success.

On the other hand we often have a case presented where the conditions appear to be in every respect favorable for successful treatment, and we exercise our best judgment and skill in treating it and yet our efforts are not crowned with success, although we are unable to ascertain any cause for the failure. Consequently we conclude that no method of manipulation is absolutely sure to result successfully.

A great mistake that is often made after capping a pulp, is in not paying particular attention to the effects of the operation. In some cases inflammation sets in after a few months, which is indicated by soreness in the tooth, and many delay until periostitis is really established before making any attempt to restore a healthy condition. This is evidently wrong; the filling should be at once removed and the pulp relieved. Sometimes this can be done, and, by the right course of treatment, the vitality still preserved in the tooth; but in a majority of cases we shall find it necessary to destroy the pulp wholly and fill to the apex of the root.

Whatever the condition of the patient who places himself under our care, it is a duty we owe to him and to the profession, to give him the benefit of our best judgment and skill in the treatment of his case at what ever cost of time and labor. We know that many bring reproach upon the profession by careless and unskillful practice and cause needless suffering to their patients, who affirm that dentistry is a fraud and that the members of the profession are unscrupulous designing men, who receive their money without giving an equivalent in return.

While in some cases we cannot blame the individuals for holding and expressing such views, at the same time we hope that all occasion for these criticisms may be removed, and the honest, conscientious practice of the majority may put to shame the unscrupulousness of the few.

